# Meeting the Demand for Different Nitrogen Forms in Potato Plants Without the Use of Nitrification Inhibitors

**DOI:** 10.3390/plants13223177

**Published:** 2024-11-13

**Authors:** Yangyang Chen, Xiaohua Shi, Yang Chen, Jing Yu, Yonglin Qin, Liguo Jia, Mingshou Fan

**Affiliations:** 1 College of Agronomy, Inner Mongolia Agricultural University, Hohhot 010019, China; 2 Inner Mongolia Academy of Agricultural and Animal Husbandry Sciences, Zhaojun Road, Yuquan District, Hohhot 010031, China; 3 College of Resources and Environment Science, Inner Mongolia Agricultural University, Hohhot 010018, China

**Keywords:** nitrogen form, nitrogen application frequency, nitrification, potato growth stage, tuber yield, soil N residual, nitrogen use efficiency

## Abstract

The preference of potato plants for specific nitrogen (N) form changes with growth stage. Potato plants prefer nitrate N before tuber formation, while they favor ammonium N after tuber formation. However, few studies have focused on N species management in potato production. In this study, 2-year field experiments were conducted from 2020 to 2021 in Inner Mongolia, China, under drip irrigation with four N treatments: (1) CK (no N was used), (2) conventional farming practices (F) (urea was the only N source applied for potato growth), (3) nitrate N supplied before tuber formation and ammonium N with nitrification inhibitor supplied after tuber formation (N-NI), and (4) nitrate N supplied before tuber formation and frequent, low-dose ammonium N applied after tuber formation (Opt). The results demonstrated that, compared with the F, the Opt treatment facilitated potato N uptake, with a 33–40% increase in plant N accumulation, and significantly increased potato growth, which ultimately resulted in a yield increase of 12–20% and an increase of 11–22 percentage units in NUE. In addition, the Opt treatment reduced the soil N residual by ~14% after harvest. Compared with the N-NI, the Opt treatment did not result in a decrease in tuber yield or NUE. Therefore, supplying nitrate N before tuber formation and frequent, low-dose ammonium N after tuber formation can result in a better match between the supply and demand of potato plants for N forms without the use of nitrification inhibitors, improving both potato yield and NUE, which is of substantial agronomic and environmental value.

## 1. Introduction

Nitrogen (N) is the most important nutrient for crop growth and yield. Accordingly, crop growth and yield can be improved substantially with sufficient N fertilizer supply, especially in less fertile soils. However, excessive application of N that does not contribute to enhanced yield or quality returns is an economic loss and has negative environmental impacts [1]. Thus, proper N management is particularly important.

Potato (*Solanum tuberosum* L.), the fourth most important food crop worldwide following corn (*Zea mays* L.), wheat (*Triticum aestivum* L.), and rice (*Oryza sativa* L.), plays a significant role in developing countries to cater to the rapidly increasing demand for a food source with the accelerated rate of population growth [2]. China ranks first in the world for the potato production area. Potato requires a relatively large amount of nitrogen, with approximately 5 kg of N required to produce 1 t of tubers [3]. However, potato has a relatively shallow root system with low root density [4,5], which results in low nitrogen use efficiency (NUE). For example, in Inner Mongolia, one of the most important potato production regions in China, the NUE is only approximately 30% [6]. These factors drive researchers to investigate ways to optimize the N fertilization rate and N application time to improve NUE in potato production [7,8,9,10]. However, few studies have focused on N species management in potato production, although the preference of potato plants for N form was reported as early as in the 1980s [11].

Nitrate (NO_3_^−^) and ammonium (NH_4_^+^) are the forms of N taken up by plants. However, many crop species have a clear preference for N form [12,13,14], and the same crop can have even different preferences for N forms at different growth stages [13,14,15]. Previous studies under sand culture conditions have revealed differences in N form requirements during different growth stages of potatoes [16,17]. Further studies under pot cultivation have shown that nitrate N is preferred by potato before tuber formation, whereas ammonium N is preferred after tuber formation [18].

The nitrate N preference of potato plants before tuber formation can be easily satisfied by the application of nitrate N fertilizer at sowing. However, a single application of ammonium N fertilizer under field conditions cannot meet the requirement for ammonium N in potatoes after tuber formation, as the ammonium N fertilizer applied to soil can easily be converted into nitrate N during this period through the soil nitrification process [19]. Nitrification inhibitors can effectively reduce nitrification in agricultural ecosystems, reduce the leaching loss of N fertilizer and improve NUE; however, certain challenges exist in practical application, such as potential toxicity, environmental pollution and other problems [20,21,22,23]. Owing to concerns about the negative effects of nitrification inhibitors on the soil, the requirement for ammonium N in potato after tuber formation is not satisfied in potato production.

As more than 60% of the total N required is absorbed by potato plants after tuber formation [3], satisfying the demand of potato plants for ammonium N is of great agronomic importance. Thus, it is necessary to explore a practical method that does not involve the use of nitrification inhibitors to meet the requirements of potato plants for ammonium N after tuber formation.

We hypothesized that the frequent, low-dose application of ammonium N after tuber formation may reduce the percentage of ammonium N that is nitrified, thereby improving potato ammonium N supply. The objective of this study was to test this hypothesis by conducting 2-year field experiments under drip irrigation with four N treatments (conventional urea, ammonium N with a nitrification inhibitor, and frequent, low-dose ammonium N application) from 2020 to 2021 in Inner Mongolia, China, and to compare the three treatments in terms of potato N absorption, plant growth, tuber yield, NUE, and soil N balance.

## 2. Results

### 2.1. Potato Yield

The results (Table 1) over two years showed that the potato yields ranged from 34 to 55 t ha^−1^, with a percentage of 53–81% for the commercial tuber rate. Compared with conventional farming practices (F), the Opt treatment increased the total tuber yield by 12–20% (*p* < 0.05), whereas no significant difference was detected between the N-NI and Opt treatments (*p* > 0.05). The commercial tuber rates under Opt were also significantly higher than those under F (*p* < 0.05). In the two years, both the total yield and the commercial tuber rate were the lowest under CK (*p* < 0.05).

According to the prices of commercial potatoes, fertilizers and other input prices in the experimental year, the net incomes of different treatments were calculated. The results showed that, compared with the F model, the Opt and N-NI increased the net income by 8435–9510 RMB Yuan ha^−1^ (Table 1).

### 2.2. Leaf Area Index

**Figure 1:**
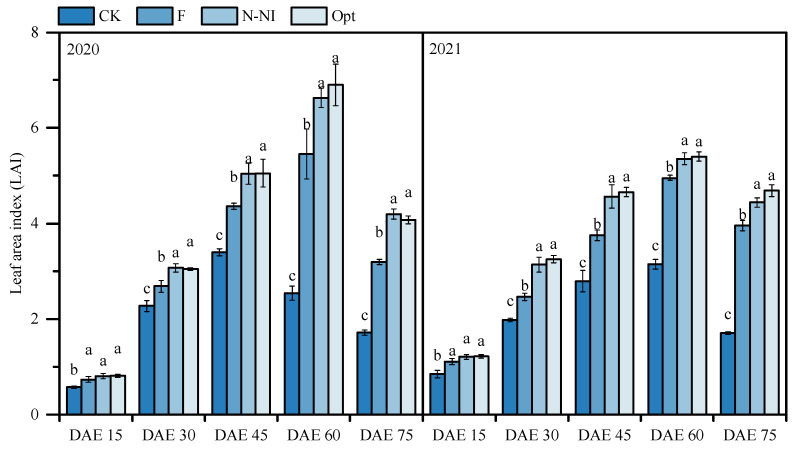
Leaf area indices of potato plants under different N management practices from 2020 to 2021. DAE: days after emergence. The column and bar in the figure represent the LAI mean and standard deviation for each treatment. Data with different lowercase letters indicate significant differences between treatments at the same period within the same year (*p* < 0.05).

Figure 1 shows that the LAI increased with potato growth and reached a maximum at the starch accumulation stage for all the treatments in all years. Under Opt and N-NI, the LAI was 2.7–16.3% greater than that under F at each growth stage, and the LAI under CK was consistently the lowest (*p* < 0.05), while no significant difference was detected between N-NI and Opt in terms of the LAI (*p* > 0.05). The same trend was observed over the two years.

### 2.3. Plant Dry Weight

**Figure 2:**
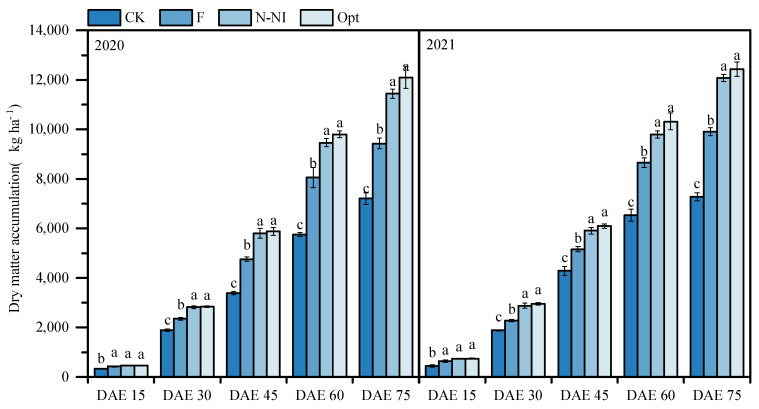
Effects of different nitrogen management practices on dry matter accumulation in potato (kg ha^−1^). DAE: days after emergence. The column and bar in the figure represent the DW mean and standard deviation for each treatment. Data with different lowercase letters indicate significant differences between treatments at the same period within the same year (*p* < 0.05).

At 15 DAE, no significant difference in plant dry weight (DW) was observed among F, N-Ni and Opt. From 30 DAE to 75 DAE, the DW of potatoes under Opt was significantly greater than that under F and CK, and the difference among treatments increased with potato growth. For example, at 30 DAE in 2020, the DW under Opt was 18% greater than that under F, whereas at 75 DAE, it was 23% greater than that under F (*p* < 0.05). No significant difference in DW was observed between Opt and N-NI (*p* > 0.05). The DW was lower in the CK treatment than in all the other treatments at each growth stage (*p* < 0.05) (Figure 2). The results showed similar trends over the two years of the experiment (Figure 2).

### 2.4. Plant N Accumulation and N Use Efficiency

**Figure 3:**
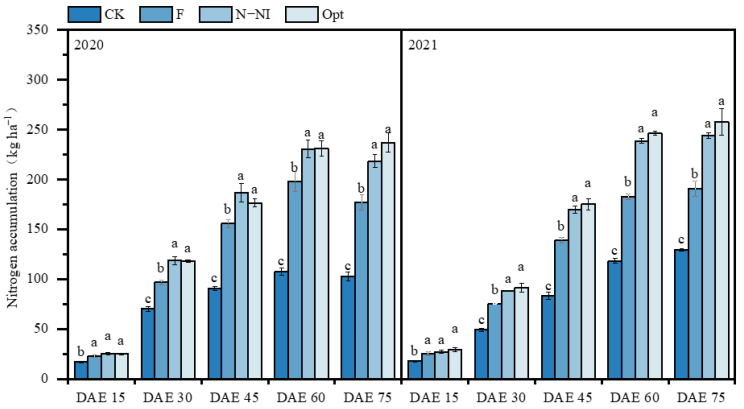
Effects of different N management practices on N accumulation in potato plants (kg ha^−1^). DAE: days after emergence. The column and bar in the figure represent the N accumulation mean and standard deviation for each treatment. Data with different lowercase letters indicate significant differences between treatments at the same period within the same year (*p* < 0.05).

Figure 3 shows the potato N accumulation at each growth stage under different N management practices over two years. Similar to DW, plant N accumulation under F, N-Ni and Opt was not significantly different at 15 DAE (*p* > 0.05), whereas from 30 DAE, plant N accumulation under N-NI and Opt was significantly greater than that under F, and the difference among the treatments increased with potato growth (*p* < 0.05). At 75 DAE, the plant N accumulation under Opt was 33% to 44% greater than that under F over the two years. There was no significant difference in plant N accumulation between Opt and N-NI at each growth stage (*p* > 0.05), while the plant N accumulation under CK was the lowest during potato growth. A similar trend was observed in both years (Figure 3).

The nitrogen use efficiency (NUE) values under the different treatments are listed in Table 1 The NUE under Opt ranged from 40% to 42% over the two years, which was significantly greater than that under F (*p* < 0.05). No significant difference was detected in NUE between N-NI and Opt in any year of the experiment (*p* > 0.05).

### 2.5. Soil N Residual

The plant removal N and soil residual N after harvest under different N management practices is shown in Table 2. Over the two years, the amount of N removed by plants under Opt ranged from 237 to 255 kg ha^−1^, which was approximately 34% higher than that under F. After harvest, the amount of residual N in the soil under Opt was 14% less than that under F. Under CK, the soil residual N amount was the smallest. No difference was observed in the soil residual N between Opt and N-NI (*p* > 0.05).

## 3. Discussion

As potato is relatively inefficient in N uptake [4,5], many researchers have been investigating ways to optimize the N fertilization rate and N application time to improve NUE in potato production [7,8,9,10]. However, few studies have focused on N forms management in potato production. The results in this study clearly demonstrated that, compared with the N-NI treatment, the Opt treatment did not result in a yield reduction over the two years (Table 1). This suggests that simply increasing the ammonium N fertigation frequency from 2 to 4 while maintaining the total N applied can have an effect similar to that of nitrification inhibitors, improving the match between ammonium N supply and demand in potatoes. Compared with the conventional N management mode F, Opt increased the tuber yield by 12–20% and the commercial tuber rate by 8–11 percentage points over 2 years (Table 1). This finding confirms the report by Suyala et al. [18] and suggests that the Opt mode could be a way to implement the N management strategy of “nitrate before tuber formation and ammonium after tuber formation” in potato production without the use of nitrification inhibitors. This was strongly supported by the results showing that the plant population under Opt consumed 33% to 44% more N than that under F did over two years (Figure 3), which resulted in a significant increase in NUE for Opt compared with F (Table 1). Thus, employing the Opt mode can not only enhance nitrogen use efficiency in the potato production system but also avert the environmental issues caused by the utilization of nitrification inhibitors.

The tuber bulking and starch accumulation stages are critical periods for potato yield, and the LAI during these periods is closely related to the tuber yield [3]. The 2-year results of this study clearly revealed that, from the tuber formation stage to preharvest, the LAI under Opt and N-NI was consistently greater than that under the conventional mode F (Figure 1). As the light interception and photosynthetic assimilation capacity of the potato canopy correlates with LAI closely [3], the higher LAI during critical growth periods laid the foundation for improved plant growth (Figure 2) and ultimately increased yields under Opt and N-NI (Table 1).

Nitrate leaching is a major route for N fertilizer loss from fields, especially from those with sandy textured soil. For example, in Inner Mongolia, more than 17% of the N fertilizer supplied to potato fields is lost via leaching, causing environmental risks, as the nitrate concentration in groundwater near potato farmland can reach 87.4 mg NO_3_-N L^−1^, far exceeding the threshold of China’s drinking water standard of 20 mg NO_3_-N L^−1^ [24]. Since most of the colloids in the soil are negatively charged, and the ammonium ion is positively charged, this leads to the adsorption of ammonium ions by the colloids. Therefore, compared with nitrate N fertilizer, ammonium N fertilizer is less expensive and less prone to leaching. This implies that the frequent, low-dose supply of ammonium N via drip irrigation after tuber formation can not only meet demand of potato plants for N form and reduce production costs but also reduce environmental risks from N leaching. This is supported to some extent by the soil residual N results, in which the soil residual N under Opt over two years was ~14% lower than that under F (Table 2).

In semiarid potato production regions, such as Inner Mongolia, China, drip irrigation is increasingly used, and frequent irrigation with small amounts of irrigation water is recommended to improve both water-use efficiency and fertilizer use efficiency [25,26]. Thus, the Opt mode fits the irrigation strategy well in potato production, and it can be conveniently implemented, which increases its agronomic value.

## 4. Materials and Methods

### 4.1. Site Description

**Figure 4:**
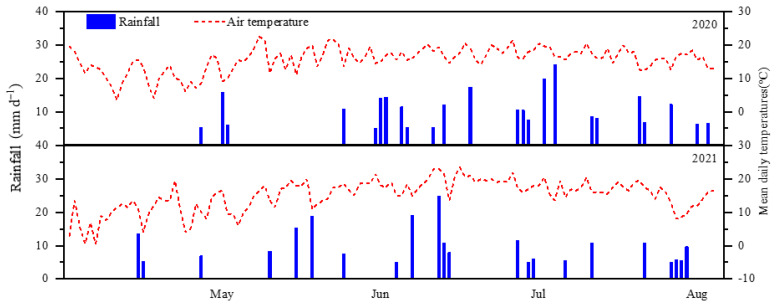
Precipitation and mean daily air temperatures during the two years of the experiment.

The experiments were conducted in Inner Mongolia (41°30′ N, 111°64′ E) in China in 2020 and 2021. The detailed physical and chemical properties of the field soils at the 0–20 cm depth are reported in Table 3. Soil texture was sandy loam. Precipitation and mean daily air temperatures in experimental years are shown in Figure 4. The precipitation during potato growth was 185.9 mm in 2020, and 137.3 mm in 2021. The previous crops were wheat in the two years.

### 4.2. Experimental Design

Four N treatments under drip irrigation condition were established for the experiment: (1) no nitrogen (CK); (2) conventional farming practices (F), in which urea was used as the N source, 30% of the total N was applied at sowing, and the remainder was supplied fertigation at 20 and 40 days after emergence (DAE); (3) nitrate first, and then ammonium with nitrification inhibitor (N-NI), in which nitrate N (30% of total N) was applied at sowing, ammonium N (70% of total N) and nitrification inhibitors were fertigated at 20 and 40 DAE, respectively, and the nitrification inhibitor was dicyandiamide (DCD), where the dosage was 5% of the total ammonium N fertilizer; and (4) frequent, low-dose ammonium N application (Opt), in which nitrate was applied as described in the N-NI treatment before tuber formation, and after tuber initiation, ammonium N was drip-fertigated every 10 days. The details are presented in Table 4. The nitrate source was calcium nitrate, and the ammonium source was ammonium sulfate. The total amount of nitrogen fertilizer applied for each treatment was 300 kg N ha^−1^. This amount is widely recommended in the target region of 45–50 Mg/ha. Phosphate fertilizer (P_2_O_5_ 180 kg ha^−1^) and potassium fertilizer (K_2_O 270 kg ha^−1)^ were broadcast at sowing. For nitrogen fertilizer, 90 kg N ha^−1^ was broadcast at sowing, while the remainder for each treatment was applied through drip fertigation as per Table 4. Generally, potatoes are drip-irrigated 10 times during the growth period, with 30 mm of water each time. During implementation, the irrigation amount is adjusted empirically according to the rainfall at each growth stage. The total irrigation amount was 235 mm in 2020, and 310 mm in 2021. Thus, the total water supply during the potato growth period was 420.9 mm in 2020 and 447.3 mm in 2021, respectively. A completely randomized block design with 3 replicates was adopted for the experiment. Each plot had an area of 90 m^2^ and contained potato plants in rows 90 cm apart, with 24 cm between plants within a row, resulting in a planting density of 46,300 plants ha^−1^. The cultivar Kexin-1 was used, its growth duration is approximately 95 days. The seed tubers were provided by Saifeng Seed Potato Propagation Company, Hohhot, China. In 2020, planting was performed on 8 May, and the harvest was conducted on 10 September. In 2021, planting was performed on 5 May, and the harvest was conducted on 3 September.

### 4.3. Soil Sampling and Measurements

Before sowing and after harvest, 9 soil samples were collected in each plot at depths of 0–60 cm and divided into three layers: 0–20 cm, 20–40 cm and 40–60 cm. All the soil samples were used to determine soil nutrient content and basic physicochemical traits. Soil ammonium and nitrate N were extracted with a 2 mol L^−1^ KCl solution and measured via spectrophotometry with a continuous flow analyzer (SKALAR SAN++, Breda, The Netherlands). The soil total N was analyzed using H_2_SO_4_-H_2_O_2_ digestion and Kjeldahl N determination (K1100, Hanon, Jinan, China). The soil available phosphorus was measured using 0.5 mol L^−1^ Na HCO_3_ extraction and spectrophotometric determination (Alpha1500, LASPEC, Shanghai, China). The soil available potassium was analyzed using 1 mol L^−1^ NH_4_OAc extraction and flame photometric determination (Sherwood, UK). The soil organic matter was measured according to Bao [27]. In this method, with external heating at 170–180 °C, the potassium dichromate—sulfuric acid solution was utilized to oxidize the soil organic matter. Subsequently, the remaining oxidizing agent was subjected to titration with ferrous sulfate, and the content of organic carbon was calculated accordingly.

### 4.4. Plant Sampling and Measurements

Plant samples were collected at 15, 30, 45, 60, and 75 DAE, and four potato plants were randomly selected from each plot at each sampling time. The plant samples were collected from the roots, stems, leaves and tubers. After the fresh weight of each part of the plant was measured, the leaf area was measured using a plant leaf scanner (LA-S, Hangzhou, China). Each sample was subjected to enzyme inactivation in an oven at 150 °C for one hour. Subsequently, it was dried at 80 °C to a constant weight for subsequent weighing and nitrogen analysis. Each dry sample was ground and passed through a 0.25 mm sieve, after which the total N concentration of the sample was determined spectrophotometrically via a continuous flow analyzer system (SKALAR SAN++, The Netherlands).

At the end of each experiment, potatoes in 30 m^2^ of each plot were harvested, and the tuber yields were reported in Mg ha^−1^. Tubers weighing 150 g or more were classified as commercially valuable tubers, hereafter referred to as commercial tubers.

### 4.5. Statistical Analysis

All experimental data were categorized, recorded, and processed via Excel 2010. Two-way analysis of variance (ANOVA) was performed via SPSS 25.0 software (IBM SPSS version 25.0, Chicago, IL, USA), and a least significant difference test was used for multiple comparisons. Graphs and figures were generated via Origin Pro 2019 software (Origin Lab Corporation, Northampton, MA, USA) and Microsoft Excel 2010 software.

### 4.6. Calculation

Nitrogen use efficiency (NUE) (%) was calculated via the following equation:

$$NUE\left(\%\right)=100\times ((N_{N}-N_{CK})/FN)$$

where N_N_ represents N uptake by potato plants in an N-fertilized plot (kg ha^−1^), N_CK_ represents N uptake by potato plants in a plot not fertilized with N (kg ha^−1^), and F_N_ represents the nitrogen fertilizer application rate (kg ha^−1^).

The leaf area index (LAI) was calculated as:

LAI=LA/I

where LA is the total leaf area of the plants sampled and L is the area of the land in which the sampled plants grew.

## 5. Conclusions

The results of this study clearly demonstrated that, compared with conventional N management practices (F), supplying a nitrate N source prior to tuber formation, along with frequent applications of ammonium N source at low doses after tuber formation (Opt) facilitated potato N uptake, with a 33–40% increase in plant N accumulation, and significantly increased potato growth, which ultimately resulted in a yield increase of approximately 12–20% and an increase in the NUE of 11–22 units in %. In addition, it reduced the soil N residual by ~14% after harvest. Compared with the N-NI treatment in this study, the Opt treatment did not result in a decrease in tuber yield or NUE. Therefore, the frequent, low-dose application of ammonium N after tuber formation can match the demand of potato plants for ammonium N to some extent without the use of nitrification inhibitors, thereby improving potato yield and NUE, which is of greater agronomic and environmental value. In the future, it is worthy of further exploration to establish the application technical parameters on soils with different fertility levels so as to achieve better application.

## Figures and Tables

**Table 1:** Tuber yield, net income and N fertilizer use efficiency of potatoes under different N management modes.

Year	Treatment	Yield t ha^−1^	Commercial Tuber Rate %	Nitrogen Use Efficiency %	Net Income RMB Yuan ha^−1^
2020	CK	34.52 ± 1.83 c	53.35 ± 2.72 c	-	−4667.22
	F	48.54 ± 1.29 b	71.87 ± 3.20 b	24.81 ± 3.44 b	14,458.79
	N-NI	53.90 ± 1.99 a	81.31 ± 0.88 a	38.88 ± 2.25 a	23,211.21
	Opt	54.65 ± 1.49 a	81.40 ± 0.37 a	42.54 ± 1.40 a	23,969.32
2021	CK	38.73 ± 1.18 c	53.36 ± 0.77 c	-	−3024.94
	F	49.23 ± 0.43 b	62.74 ± 1.39 b	28.52 ± 1.90 b	7732.18
	N-NI	54.61 ± 1.61 a	72.79 ± 0.68 a	36.72 ± 3.26 a	16,167.22
	Opt	55.03 ± 1.44 a	73.75 ± 2.05 a	40.17 ± 1.18 a	16,552.43

The data in the table represent the mean ± standard error. Different letters after each column indicate significant differences at the 0.05 level, and differences were compared only between treatments in the same year.

**Table 2:** Plant removal N and soil residual N after harvest under different N management practices in 2020 and 2021.

Year	Treatment	Plant Removal N kg ha^−1^	Soil Residual N kg ha^−1^
2020	CK	103	54
	F	177	135
	N-NI	218	121
	Opt	237	115
2021	CK	110	71
	F	191	153
	N-NI	249	138
	Opt	255	131

**Table 3:** Physicochemical properties of the 0–20 cm soil layer in the experimental fields.

Year	OM g kg^−1^	NO_3_^−^-N mg kg^−1^	NH_4_^+^-N mg kg^−1^	Available P mg kg^−1^	Available K mg kg^−1^	pH Value	Bulk Density (g cm^−3^)	Field Water Capacity (%)
2020	23.6	7.80	0.90	13.7	129.5	8.1	1.37	24.37
2021	25.1	6.91	2.58	14.2	132.3	8.1	1.45	20.06

**Table 4:** N fertilizer input and management practices for each treatment from 2020–2021.

Treatment	Total N (kg N ha^−1^)	Sowing		Drip Fertigation				
		N Rate (kg N ha^−1^)	N Form	Rate (kg N ha^−1^)				N Form
				20 DAE	30 DAE	40 DAE	50 DAE	
CK	0	-	-	-	-	-	-	-
F	300	90	CO(NH_2_)_2_	120		90		CO(NH_2_)_2_
N-NI	300	90	NO_3_^-^	120		90		NH_4_^+^+DCD
Opt	300	90	NO_3_^-^	60	60	45	45	NH_4_^+^

DAE: days after emergence.

## Data Availability

The data supporting the results reported in the article can be made available by Yangyang Chen upon reasonable request.
